# Maddening Post-Qualitative Inquiry: An Exercise in Collective (Mad) Theorising

**DOI:** 10.1177/10497323241231896

**Published:** 2024-03-12

**Authors:** Aimee Sinclair, Lyn Mahboub

**Affiliations:** 1 School of Allied Health, 1649 Curtin University, Perth, WA, Australia

**Keywords:** post-qualitative, Mad studies, affect, mapping

## Abstract

Both post-qualitative inquiry and Mad methodologies sit on the fringes of qualitative health research, although their potential for creating new knowledges and practices is increasingly recognised. In this article, we explore the possibilities created by bringing these approaches together within research led by, or centring, mental health service users and survivors. We outline and reflect on a workshop undertaken with peer support workers to map affective intensities within mental health assemblages. We suggest the tensions between post-qualitative and Mad research approaches hold potential for mental health research, and qualitative health research more broadly, bringing together theory and the experiences of service users/survivors to think–feel–become otherwise in relation to health care, peer support, and activism.

## Introduction

What possibilities for thought and political action might open up if post-structural theories were to make their way into the kitchen table conversations and the protests, and what conceptualizations that we don’t yet have the language to describe might emerge? (Bivens, 2021, p. 399)

Conventional humanist qualitative methodologies, centring the human subject as “the ontological and epistemological hub of thought and discourse,” have historically excluded those deemed “mad” from knowledge production (Dillard-Wright et al., 2023, p. 2). With the aim of being more just, service users/survivors are increasingly included within mental health research, policy-making, and practice. Often involving a re-positioning of service users/survivors as having an authentic voice, expertise, and capacity, such approaches remain predominantly loyal to the humanist subject, despite the limitations of doing so. Within mental health research, for example, humanist methodologies often fail to attend to how practices and subjects are entangled within psychology (and other) assemblages, thus fixing subjects and practices in place, papering over difference, and neglecting the effect of both discursive *and* material relations in shaping ‘lived experience’ voices. Individuals with ‘lived experiences’ often hold an untenable position within such projects, required to perform authenticity and representation (Tseris et al., 2022; Voronka, 2016a). Despite liberatory intentions, much ‘lived experience’ research continues to psychiatrise knowledge, failing to foster deeper transformation (Brown & Jones, 2021; Rose & Kalathil, 2019; Rose, 2017; Russo, 2022).

Both post-qualitative inquiry (PQI) and Mad methodologies work to displace humanism. PQI, unable to “be tidily described,” involves a “running away” from “what is usually thought of as method,” prioritising instead thinking with theory in ways that accommodate post-humanism, accounting for multiplicity, relationality, and becoming otherwise (Lather, 2013; p. 635, Marcus, 2009, p. 6). Similarly, Mad approaches to research, with a strong focus on epistemic justice and activism, subvert the disciplining nature of conventional qualitative methodologies, centring Mad ways of thinking–feeling that have so often been cast out of academia (LeFrancois & Voronka, 2022).

In this article, we consider the productive possibilities of bringing post-humanist theorising, operationalised through PQI, to “the (Mad) kitchen table conversations and the protests” (Bivens, 2021, p. 399). That is, we consider the “possibilities for thought and political action” beyond humanist logics produced by making post-humanist theorising accessible and useful to Mad projects, both within and outside of academia (Bivens, 2021, p. 399). Simultaneously, we consider the potentials of maddening PQI, thus “unsettling the dominance of the (white) academy as the hegemonic source of knowledge production” (LeFrancois & Voronka, 2022, p. 106).

To do so, we reflect on collective work undertaken with peer support workers to map affective intensities within mental health assemblages. This collective process was developed, and facilitated, through thinking with both critical post-humanist and Mad theory alongside peer worker experiences and practices.

We start our discussion with an introduction to Mad and post-qualitative approaches, before using our own research journey to exemplify the limitations of humanism that plague lived experience research. We then outline our experiment in bringing together PQI and Mad approaches. We describe the workshop design, we then reflect on what possibilities bringing these approaches together produced, and finally, we describe some of the limitations of our process. We describe our collective work not as a method to follow (to do so would go against PQI) but rather as a provocation, of what might be possible when we work with both Mad and post-humanist theories. Similarly, rather than share ‘findings’ from our work, our focus instead is on demonstrating how collectively thinking *with* Mad and post-humanist theories, through a Mad, post-qualitative approach, may prompt new possibilities for thought and political action.

Some notes on language before we proceed: as the primary author and researcher, ‘I’ (Aimee) write in first person. I do so to attend to how my experiences, desires, abilities, and privileges shape the research and thus the knowledge produced. However, I also think–feel–write as part of a research team (Lyn, others), and thus ‘we’ feature in the paper. ‘We’ are also part of a larger assemblage of material–discursive relations (ethical boards, funding requirements, methods, tools, theories, Mad activists, and, and, and …) that produce the thinking–feeling for this paper. We use the term ‘Mad’ in a reclaimed and non-essentialist sense, resisting the ways in which certain individuals and communities come to be constructed as ‘mentally ill’, as well as to celebrate Mad ways of thinking–feeling–becoming. We recognise the politics and language of Madness does not resonate for everyone who has experience navigating (or avoiding, escaping) mental health systems, including potentially those that contributed to the collective work. Although we (Aimee and Lyn) currently identify as thinking–feeling–acting madly, we continue to grapple with the possibilities, limits, and privileges of doing so.

## Maddening ‘Lived Experience’ Research

Mad studies, centring the experiences, theorising, and activism of consumers, survivors, or ex-patients (C/S/X) of mental health systems, are a relatively new area of study (Beresford & Russo, 2021; LeFrançois et al., 2013). However, it builds on a long history of community activism and cultural work undertaken by those deemed ‘mad’ (and those acting in solidarity). Mad studies critiques the dominance of psychiatric knowledges and practices, highlighting violences inflicted through such practices, and psy-entanglements with other violent forces including racism, sexism, homophobia, transphobia, ableism, and classism (Aho et al., 2017; Cohen, 2014; Fernando, 2017; Golightley, 2023; King, 2016; LeFrançois, 2013; Mills, 2017; Ross & Costa, 2021). Such violences range from imposing discourses that deny social and structural causes of distress, and negating Mad people’s own explanations, through to ‘treatments’ with harmful effects including restraint, seclusion, drugs, and ECT (Daya, 2022; LeFrancois & Peddle, 2022).

In terms of methodology, Mad studies turns away from “studying down” onto “problemetised bodies” (Voronka, 2016a, p. 212), focusing instead on the oppressive effects of systems and practices, and recognising “potential in the so-called rants and raves of madpeople” (Bruce, 2021, p. 306). Mad methodologies are deeply concerned with the ethics of knowledge production, recognising the ways in which research often perpetuates epistemic injustice through the exclusion and denigration of first-hand knowledge(s) of individuals deemed ‘mad’. Yet Mad thinking recognises that doing so involves more than including as ‘data’ stories of ‘lived experiences’ or the rarer practice of employing individuals with lived experiences to contribute to analysis. Rather, LeFrancios and Voronka (2022) propose Mad theory as methodology, alongside an “ethics of unruliness,” to “unveil and disrupt colonial ways of knowing in order to make visible ‘the unheard of’ and to allow for (racialized) mad knowledge production” (p. 115).

## Post-Qualitative Inquiry

In many ways, Mad approaches are already entangled with the thinking and doing of PQI. Defining PQI is slippery, as St. Pierre (2019) notes, the question of what PQI *is* often remains lingering, predominantly because it is defined by what it is not. Defining PQI assumes “something already exists … is stable, and so can be identified and represented,” thus denying the immanence of PQI (p. 6). Mirroring suggestions that Mad methodologies adopt an ‘ethics of unruliness’ (Bruce, 2021; LeFrancois & Voronka, 2022), PQI refuses pre-existing methods, unsettling normative ways of thinking–doing–being, and asking questions of “what counts as knowledge, what counts as ‘real’, and who has the authority” (Jackson & Mazzei, 2018, p. 717). PQI encourages researchers to “shake off exhaustive and exhausting habits of method,” including mechanistic coding, reducing data to themes, and writing up neat, replicable findings (Jackson & Mazzei, 2018, p. 717). Rather, just as LeFrancios and Voronka (2022) propose Mad theory as methodology, PQI urges a thinking *with* theories and concepts, working in new and creative ways that put aside methodology in an ongoing process of thinking and responding differently (Jackson & Mazzei, 2018). As such, there are no set PQ research practices, other than studying theory and putting it to work (St. Pierre, 2019).

Post-qualitative inquiry accommodates theories that commonly fall under the banner of post-humanist, post-structural, and material theorising, recognising that such thinking is incompatible with conventional humanist qualitative research. Whilst we recognise the multiplicity of, and tensions between, theories that fall under these banners, in general such thinking aims to decentre both the colonial emphasis on the human, as a unified agentic subject, and attend to the material world in enacting change. Such theories attend to bodies, both human/non-human (objects, spaces, time, discourses, and emotions), “situated and material and embodied and connected” (Dillard-Wright et al., 2023, p. 2). That is, rather than understanding bodies as separate, independent, fixed, or knowable, they are understood as always in a process of becoming within ever-shifting assemblages. Much of these ‘new’ ontologies echo Indigenous worldviews and practices that have always refused colonial notions of rationality and nature/culture and mind/body binaries (Arnold et al., 2021; Milroy, 2021; Todd, 2016).

## Getting Un/Stuck

Before describing our experimenting with bringing together Mad methodologies and PQI, in this next section we first consider why such an endeavour may be productive, drawing on our own research journey to exemplify the limits to humanist practices that often plague ‘lived experience’ research.

Our own research involves the politics of peer support inclusion within the Australian mental health system. Whilst recognising peer support as plural and continually becoming, peer support is predominantly enacted within mental health contexts as involving supporting another through shared lived experiences, underpinned by the values of mutuality, solidarity, and self-determination (Stratford et al., 2019). Peer support is increasingly drawn into formal systems through the emerging discipline of peer support work, whereby individuals with certain lived experiences (of navigating mental health systems, of living through/alongside experiences commonly labelled as mental illness) are employed alongside psy-professionals (social workers, psychiatrists, and psychologists). Such inclusion is predominantly constructed as universally beneficial (Sinclair, Gillieatt et al., 2023). However, peer support workers’ experiences of workplace exclusion, harm (Alvarez-Vasquez et al., 2020; Edan et al., 2021; Irwin, 2017), and claims of co-option and de-politicisation (Adams, 2020; Fabris, 2013) challenge such notions, thus prompting our interest in the possibilities and limits of peer support inclusion.

Informed by survivor research and Mad methodologies, in many ways our initial approach stretched the limits of conventional methodologies through a commitment to epistemic justice, dialogical sharing, critical theorising, reflexivity, and a commitment to social change. Simultaneously, we started in (and continue to work within) somewhat conventional qualitative territory, with our initial research questions and processes “grounded in humanist concepts of language, reality, knowledge, power, truth, resistance and the subject” (Lather, 2013, p. 635). Extending an open invitation through peer networks, we recruited 15 individuals with experience as peer support workers within the Australian mental health system for discussions about their experiences. Purposive sampling was used for diversity across gender, age, time in peer roles, community/hospital settings, and location across states. Having experience facilitating peer support myself, these discussions were envisioned as a space of mutual sharing and theorising, where both myself and fellow contributors to the research might become otherwise. I had planned to thematically code these discussions, with a focus on how dominant psy-discourses shape peer support work/ers and how peer support workers may resist (see Sinclair, Mahboub et al., 2023 for further detail).

As a Mad researcher, I felt constrained by the limits of conventional humanist requirements from the start of the project (Sinclair & Ridley, 2022). However, it was during reading–feeling my way through transcripts, and attempting coding, that fault lines within my research questions and methods of inquiry, underpinned by humanist logic, became impassable.

My first (un)sticking point: I got bored. As I attempted coding, I seemed to be replicating pre-existing knowledge. There was nothing that sparked new ways of thinking–feeling–doing. I recall one of my supervisors, as we worked through coding collaboratively, mentioning that the interviews reflected aspects of CHIME, a model for understanding the components and stages of ‘personal recovery’ (CHIME being an acronym for connectedness, hope, identity, meaning, and empowerment) (Bird et al., 2014). She was right, these themes could be extracted from the discussions. My heart sunk. Was this all I had done? Just collected examples of an already established framework. How would this extend Mad thinking and activism, as I so desired?

This feeling, I soon discovered with relief, is not unusual. Jackson and Mazzei (2018) describe the macro focus that comes with coding can create predictability and a lack of new knowledge. They describe how, during a study with first generation academic women, they could have been “good qualitative researchers” presenting major themes and patterns in writing up the findings (p. 729) Yet, these practices would have resulted in predictable knowledge because “our formulation of the categories would have been simply driven by our experience and that of our participants, devoid of any philosophically informed concepts that would jolt us out of received ways of knowing” (p. 729). Thinking with Mad theory stretched me to consider alternative ways of understanding beyond established psy-discourses. Yet, combined with mechanistic coding, I was unable to account for the complexities and difference within the transcripts and my own thinking–feeling, nor the new knowledges we were seeking. Rather, I was simply replicating existing knowledge.

Further, I noticed within our discussions a tendency to binarize and fix peer support practices and workers into ‘authentic’ and ‘co-opted’ categories. Whilst this speaks to a growing and valid concern regarding the effects of inclusionary practices, the experiences and practices peer support workers shared rarely fitted such binaries. Multiple, shifting subjectivities, experiences, and practices challenged the subject of ‘authentic’ peer support work(ers) and a notion of individual resistance. Further, peer support practices appeared to have both liberating *and* oppressive effects, as did other relations within mental health systems, thus challenging ideas of psychiatry as a monolithic and impenetrable system, and the inclusion of peer support workers as having uniformly liberatory effects (Sinclair, Gillieatt et al., 2023). Simply coding ‘lived experience’ voices regarding the mental health system (as universally bad) and peer support (as universally beneficial) papered over these differences and failed to acknowledge the unsaid.

This risk, of universalising and fixing complex practices, identities, and affects, is one that has been highlighted for Mad studies (Cresswell & Spandler, 2016; Rose, 2017). Doing so sidelines the diverse and dynamic nature of practices and subjectivities and their entanglement with other complex forces, epistemically suppressing differences across positionings and limiting the ability to imagine diverse possibilities for activism (Rose & Kalathil, 2019; Rose, 2017).

This effacing of difference was also apparent within our discussions through the largely absent recognition of the ways in which inclusion and “madness lands and is graphed on bodies differently” across class, gender, sexuality, race, and diagnosis (Jones & Kelly, 2015; Voronka, 2016b, p. 197). In Australia, for example, it is rare for work by scholars and activists from racialised communities to be discussed and acted upon as an integral part of peer support, despite vital contributions that have exposed the entanglements of colonialism, racism, and psychiatric oppression, as well as the emancipatory potential of queer/trans/Black Mad care practices (Eales & Peers, 2021; Piepzna-Samarasinha, 2018; Pilling, 2022). With peer support predominantly constructed as liberatory (Sinclair, Gillieatt et al., 2023), we rarely discussed the harm done as (predominantly white, sane-passing) peer support workers and how we all reinforce colonial, patriarchal, and capitalist effects under the banner of ‘lived experiences’. My own limitations as an interviewer from a relatively privileged position also inhibited thinking–feeling about such relations. Instead, with best intentions, we often talked of a “common humanity,” an “appeal to an idyllic oneness where difference is blithely effaced” (Bruce, 2021, p. 10). I wondered how I might attend to these differences that went unsaid within the transcripts or were being erased through coding.

There were additional (un)sticking points. Sharing with fellow peer support workers, we often experienced visceral responses and emotions: ‘tingles’, tears, frustration, despair, and joy. Such responses were absent from the transcripts or marked by ‘you knows’: a reference to shared affects without the language to articulate these. So too were my responses reading the transcripts and thinking with various theories. I often found myself feeling hopeful and energised, or tense, my insides in knots. There is no space in conventional discourse-orientated analysis to consider these. Brown and Jones (2021) suggest that when we “look to service users for ‘information’ only, we miss the more generative experience of being moved, of opening ourselves up to the stories and experiential knowledge of others, and of appreciating the force of emotion in generating change” (p. 964). For individuals who have had interactions with psychiatry (and particularly, as Rose and Kalathil (2019) point out, the racialised mad), such emotions are pathologised, a result of a dysfunctional brain or poor coping behaviours, deeming the knower irrational or untrustworthy. How might I instead value these affects, using them to push knowledge and action in new directions?

Further, material objects – case notes, goal-setting sheets, locked doors, meeting spaces, and high-heeled shoes – punctuated our discussions and shaped practices in ways that needed accounting for. There was a stark difference, for example, articulated between community and clinical spaces that could not be explained by discourse alone. Peer support workers articulated how such materiality affected the ways they thought, felt, and practiced peer support, yet again – how to attend to these relations through conventional qualitative methods? As it was, my approach was reinforcing ideas of agentic, independent peer support workers, divorced from their material–discursive entanglements, and failing to conceptualise resistance outside of an individualistic model that held peer support workers responsible for their ability to resist or be ‘seduced by inclusion’.

Engaging with post-humanist theories alongside Mad theory, peer experiences and practices, and our shared and contested affects and desires opened up ways through these sticking points. Rather than exploring the idea of ‘authentic’ peer support, I began thinking about peer support as continually brought into being through an ever-shifting assemblage of human and non-human intra-actions. Attending to peer support practices and identities in a constant state of flux, relational and contextual, I was able to put aside coding and think–feel–write my way through the transcripts differently, attending to the value of mad feelings, the material world, intra-actions, and difference. In some ways, stumbling across PQI through post-humanist theories also gave me permission to do some of what, as (Mad) researchers, we already do, but feel the need to hide within conventional frameworks (Koro-Ljungberg, 2016). For example, no longer did my thinking need to be contained to representing or interpreting the ‘data’. Rather, I had permission to think diffractively through other materials not traditionally considered ‘data’: theory, emotions, researcher perspectives, and experiences (Fox & Alldred, 2021). It was through these productive possibilities that we became interested in how we might further entangle Mad approaches and PQI in a way that might inform collective theorising and activism, both within and outside of academia. It is to this work we now turn.

## Thinking–Feeling–Becoming Together: Collective (Mad) Theorising

In our initial ethics application (Curtin University HRE2019-0152), we had included a workshop to follow the initial discussions with peer support workers. This workshop was originally conceived as an opportunity to enable Mad knowledge production through further critical co-reflection and theorising with peer support workers. The structure and content of the workshop had been purposefully (un)designed to be flexible, shaped by the initial thinking–feeling with peer support workers. The struggle often, with ethics applications, is that one must know in advance the instruments needed to investigate a field. And yet, such questions and instruments, as they did in our case, become through the *doing* of research rather than being known a priori and then applied (St. Pierre, 2019). For me, it was through sharing and theorising with peers, reading–feeling through transcripts and theory, and attempts at coding, that I became ‘stuck’, and through which something different began to emerge. We were therefore lucky to have the space to create a new way to explore the subject when our original research questions and methods no longer fitted. In this next section, we describe the process of this collective work.

Our aim was to map affective relations within the Australian mental health system, consider what these relations produce in terms of peer support, and consider how support might be enacted differently. Drawing on post-humanist theorising, we sought to move past the idea of workers as ‘pre-formed agents’, rational bodies thinking/acting in linear ways in static health care settings (Gibson et al., 2021; Stone et al., 2020). Rather, in planning the workshop, I conceptualised peer worker experiences and practices as entangled, decentring the peer support worker as a knowing, acting, and autonomous subject and attending to material relations as affective (the way in which bodies, emotions, physical spaces, and objects affect one another). The aim was thus not to represent ‘truth’ but rather to prompt thinking–feeling–doing otherwise.

All contributors to the initial discussions were invited to contribute to the workshop. Four contributed and we (Aimee and Lyn) both facilitated and contributed. All contributors were familiar with critical mental health and Mad theorising. The workshop was held online for 3 hrs, recorded, and later transcribed. Contributors were paid for the time they spent engaging with materials independently and collectively, although we recognise the small amount of funding provided does not adequately recognise the emotional and mental labour and expertise involved with such work, as is often the case in lived experience involvement (Brosnan, 2019; Faulkner & Thompson, 2021; Papoulias & Callard, 2022). Ethics approval was provided by the Curtin University Ethics Committee (HRE2019-0152), and all contributors signed consent forms. In saying this, our Mad ethical commitments extend past the minimum requirements of ethics boards to ‘do no harm’, to instead centre practices that enable Mad knowledge production (LeFrancois & Voronka, 2022). Contributors to the workshop were not considered as providing “data” or as “participants,” but rather as “theoretical provocateurs and theorists” (Liddiard et al., 2019, p. 1).

Thinking about the design of the workshop, we (Aimee and Lyn) sat with potential tensions between Mad studies and PQI. For us, one of these tensions is that the theoretical works underpinning PQI are dense and can be experienced as exclusionary. Despite having access to undergraduate sociological studies, when I first engaged with post-humanist theories, my head hurt. I struggled. As a research team, we continue to struggle. Yet at the same time, such theorising provided us with something to think diffractively with, moving us past many of the ‘sticking points’ of humanism. Given Mad studies’ firm commitment to ensuring theory, research, and grassroots political action is intimately connected (LeFrancois & Voronka, 2022), we thought carefully about how we might operationalise post-humanist theory in ways that validate and enable “madpersons as critical theorists and decisive protagonists in struggles for liberation” (Bruce, 2021, p. 9).

With these considerations in mind, I created a series of online videos for viewing prior to the workshop, based on feedback from contributors about preferred formats for information. The videos discussed the research assemblage (academic culture, my experiences, desires, family life, madness, technology, COVID, theory, transcripts, and policy) that had produced the thinking–feeling–doing for the project thus far and the theories informing the workshop design. I conceptualised my role as a quilt-maker, picking up on threads from our individual discussions and combining them with theory and my own experiences to weave a story. In speaking about theory, I acknowledged harms produced through academia and my own complex relationship with/in academia. I shared how theories have both been lifesaving to me as well as how I have often felt excluded and othered by theory. I shared how a separation of mind and body means that feelings and bodily responses are often de-valued and pathologised, and how I wanted to challenge this, seeing them instead as valuable knowledge sources. Using imagery of a rhizome, I described how certain elements in our world come together to produce ways of thinking–doing that strengthen over time, whilst other combinations may produce lines of flight, new and different ways of doing–thinking–growing.

The recordings served multiple purposes: facilitating transparency of knowledge creation; prioritising accessibility; and enabling thinking–feeling with theory separately and together.

In our planning and facilitation, we worked towards supporting a ‘safe enough’ (Reynolds, 2013) and ‘dignifying’ space (Moran & Ridley, 2021) for collective theorising. Prior to the workshop, we discussed with the other contributors how best to do this, which included providing all materials prior to the workshop. It also included dedicating a significant portion of time at the start of the workshop to collectively share our positionalities and previous research experiences, acknowledging the epistemic injustices produced through mental health research, and how we might use our experiences of such to act differently. We collectively re-visited theory and how this shaped the task for the collective work, working through key concepts and providing examples, noting that neither Aimee nor Lyn were an expert in the field, but were learning, in relation with others.

An additional point of tension for us was working through PQI’s requirement to avoid imposing a universal “moral framework” (Davies et al., 2013, p. 680), alongside tendencies within Mad theorising and activism (as with other emancipatory spaces) towards totalising narratives and identities. PQI avoids making normative statements about how things ought to be, or how one ought to act, instead opening “up a moment-by-moment ethical questioning that asks how things come to matter in the ways they do” (p. 680). It “demands of the researcher new skills of listening to the minute details of life as it unfolds in all its multiplicity, in its repetitions, and in its leaps into the unexpected and new” (p. 680). It is this moment-by-moment ethical questioning that enables a consideration of complexity and difference, to consider the ‘unthought’. Doing so does not mean researchers deny moral judgements or ontological situatedness but rather demands an attentiveness to these and a willingness to work with the “interaction and contestation between different worlds” (McLeod & Fullagar, 2021, p. 6). Yet, given many of us who are familiar with critical mental health and Mad theorising adopt a critical stance towards psychiatric dominance, and/or have experienced psychiatry as oppressive, it can sometimes be difficult to put aside moral assumptions or the adoption of universalising ideal subjects, identities, or practices (e.g. peer support = liberatory, psy-practices = oppressive). For me, I also worried about becoming ‘domesticated’ by post-humanist theories, losing my activist energy, and ability to make strong statements regarding psy-violences.

Here, I found the contributions of scholars who are already working with post-humanist theories alongside Mad, feminism, crip, and anti-colonial studies valuable (Aho et al., 2017; Blackman, 2015; Bruce, 2021; Cvetkovich, 2012; Fritsch, 2010; Fullagar et al., 2019). Bruce (2021), for example, works with Mad theory yet diverges from a Mad studies “steadfastly arrayed against biomedical psychiatry” (LeFrançois et al., 2013, p. 13). Whilst “decry[ing] the dire harm that biomedical psychiatry has wrought on many pathologized people,” they suggest that a validation of all experiences means “we might sometimes cautiously, provisionally, ambivalently, improperly, subversively take up biomedical psychiatry—all while we pursue its radical transformation” (p. 14). Suggesting an already entanglement of madness and PQI, Bruce (2021) adopts a Mad methodology that “seeks, follows, and rides the unruly movements of madness,” that values the “rants” and “silences” and mad feelings that defy reason, that historicises and contextualises madness, cultivating “critical ambivalence to reckon with the simultaneous harm and benefit that may accompany madness” (p. 9). Thus, I considered how we might be “both/and” in our collective thinking–feeling–doing research; sensitive to psy-violences, whilst also open to a moment-by-moment ethical questioning; “refusing a rush to interpret (or interrupt)”; and allowing ourselves to be moved by difference (Pillow, 2019, p. 120).

Operationalising such considerations, during our initial discussions, we acknowledged tendencies that might inhibit our collective theorising and how we might address these. These included the tendency to attribute sole agency to the humanist subject, judging others or specific practices based on pre-existing moral frameworks, the desire to empathise with one another’s experiences and seek connection and recognition (thus papering over difference, dropping into normativity, and failing to challenge our own understandings), and the tendency to engage in meaning-making/interpretative work based on pre-existing grand narratives. We discussed how we might feel safe to raise such tendencies when we noticed them in ourselves or others and strategies we may employ to sit with uncomfortable affects produced through being in relation with peer perspectives and practices that challenged our own, recognising these for their productive potential.

Our noticing and theorising was split across two focus areas: ‘spaces’ and ‘boundaries’, both drawn from my initial thinking–feeling with peer support workers and theory. Through thinking with feminist new materialism, my attention was drawn in the initial discussions to how the physical location of peer support and objects (clipboards, iPads, name badges, and forms) affected the way support was enacted. To explore this materiality more fully, I chose images of spaces where peer support work is commonly practiced in Australia: a hospital room within a mental health inpatient unit and a coffee shop, inspired by similar approaches to addressing materially within assemblages (e.g. Brice et al., 2021; Van de Putte et al., 2018). For the second area: boundaries, I chose anonymised quotes from the initial discussions. Boundaries as a concept and practice within mental health contexts provoked ‘affective intensities’ within our discussions, deeming the concept worthy of further exploration (Ringrose & Renold, 2014).

Alongside these images and quotes, I developed questions that supported ‘afflexivity’ (Setchell et al., 2021) and ‘mapping’ (Clarke, 2005). Afflexivity refers to “a process of intentionally reflexively foregrounding affect and emotion in a post-human intra-action” (Setchell et al., 2021, p. 2). Importantly, afflexivity is not an attempt to get closer to ‘truth’ but rather to provide a point of diffraction, honouring the productive possibilities of feelings for activism. Mapping, as conceived by Clarke (2005), involves attending to the human and non-human elements of an event, their relations, and potentials. This process of relational mapping differs from the practice of Mad mapping originating from within Mad activist, mutual aid, and healing justice circles (The Icarus Project, 2015). Rather than assuming elements as essential, stable, and universal, within relational mapping are considered “open systems that are contingent, unpredictable, and productive” (Martin & Kamberelis, 2013, p. 678). Mapping enabled us to consider both the dominant, normalising forces at play and potential for rupture. In developing the prompts, I attended particularly to the mapping of material relations, asking about smells, sounds, and objects that mattered. We moved through each of these prompts, using first the images and then the quotations, to facilitate our discussion.

## Bringing Together Madness and PQI: Possibilities

In this section, we consider the possibilities produced through our coming together (an assemblage of peer memories, Mad and post-humanist theorising, embodied responses, technology, discourses, time, and, and, and …). Rather than share ‘findings’, our focus is on demonstrating how thinking *with* Mad and post-humanist theories, through a Mad, post-qualitative approach, may prompt new possibilities for thought and political action. We share here tentatively and sitting with ethical discomfort, given the authorship of this paper does not reflect all workshop contributors. We cannot speak to what the workshop has *done* in terms of others’ thinking and activism, other than what was discussed within, and transcribed from, the workshop. As such, we focus particularly on what the workshop produced regarding our own thinking, feeling, and activism. Where we have used quotes, we have chosen to experiment with not identifying these as belonging to individual contributors, recognising such thinking–feeling as a product of material–discursive relations.

### Embracing Affect

Despite often being understood as mundane, un-affective spaces and practices, the images and quotes produced strong visceral reactions. Responses, particularly to the image of the clinical space, included breathlessness, apprehension and fear, tension and contraction, adrenalin, and disconnection from the body – a numbing; bodies stiffening; voices wavering or speeding up; and reverberations in throat:

I feel tense. Like I’ve tensed up. I feel hesitant. I feel hesitant to enter. And tense, apprehensive, tense. I guess my heart is beating a bit faster ... It’s almost like a freeze response that is stopping me.

The affects of being in this space as a peer support worker were intimately intertwined with that of having also been a ‘patient’, yet at the same time we noted difference related to these identities as they are made and unmade across encounters:

I’m having quite a reaction because I’ve stayed in that room …. at the moment that’s my past room, it’s sort of my present room because we are in a zoom room looking at it, and potentially that’s my future room.

I actually have different bodily responses when I’m reflecting on being in that room as a patient – my body kind of drops – like I get deflated, a sense of resignation. But then my experience as a peer worker was that I kind of felt … my body stiffen ... on guard.

Collectively embracing affect pushed us past reflection, as an inner mental activity. Rather than stepping back and reflecting on “the world at a distance” (Barad, 2007, p. 87), we attended firstly to the event of our intra-action with the image, how it “hits us” or “invades us” (Colebrook, 2002, p. 3). This ‘diffractive seeing’ is not limited to the gaze of the eye, or understanding an image as mirroring the world, but rather activates all bodily affective perceptions (Hultman & Lenz Taguchi, 2010). These perceptions moved us to feel–think differently, to differentiate. Our breathlessness, rapid heartbeats, and feelings of being on guard, provided an obstacle to our thinking about spaces, peer support, and human agency: an “apparatus of diffraction” (Barad, 2007, pp. 74–75). Rather than such spaces and practices as being mundane and un-affective, we came to see their affective potential, with our attention drawn to the diverse affects and effects produced through ever-shifting assemblages of forces (desires, objects, spaces, and temporalities).

The collective noticing and sharing of such responses also enabled an imagining of our bodily responses and emotions as relational: affects of broader socio-material assemblages in which we are all entangled, albeit in different ways. This is particularly pertinent for peer support workers given such responses are often enacted as a problem located within the peer support worker. Peer support workers, for example, are often produced as having unsuitable dispositions or skills for mental health work given embodied experiences of distress are produced as mental illness (and therefore a liability) rather than a result of inclusion within a clinical assemblage (Sinclair, Gillieatt et al., 2023). Feeling collectively with Mad and post-humanist theories, these affects, the hauntings of our past admissions and experiences as well as our current experiences, were not a liability but rather vital in mapping material–discursive relations that produce such affects and how we might become otherwise through such relations. As so eloquently captured by Bruce (2021, p. 14), “‘mad’ feelings like obsession and rage [can be harnessed] as stimulus for radical thought and action.”

As an example, collectively embracing affect provoked, for me, new “possibilities for thought and political action” (Bivens, 2021, p. 399). The coffee shop can be seen as representative of a community orientation, where people may escape psy-violences of the clinic. Within a hospital room, one is positioned as either a ‘provider’ or a ‘patient’, yet within a coffee shop, one can potentially be, as one peer remarked, “just out for coffee, like what people do.” Atmospheres of sociality “animated by forces of connection, interaction, expression, solicitation and concern” are often present in places like cafes, libraries, and parks and may provide a practical means of achieving recovery (Duff, 2016, p. 67). In my own life, venturing down to my local coffee shop is a vital part of my becoming-well. Drawing from my own lived experiences, I was expecting the image of the coffee shop to produce liberatory affects.

I had yet to fully conceptualise such spaces as an assemblage, and thus the materiality of the coffee shop alone does not create an affective atmosphere of recovery. Rather, it is a “unique set of encounters,” entangled in a particular way that produces connection (Duff, 2016, p. 68). The embodied responses of others brought this thinking to bear. Others described distraction, being overwhelm, irritation, energetic drain, noise, a sense of physical ‘tightness’, not having money to pay for coffee, the ‘intimidation’, and ‘self-consciousness’ of being in a ‘normal place’ where people are ‘wearing pretty clothes’, looking ‘like normal people that have got it together’. Thinking–feeling collectively highlighted the socio-economic, class, and neuro-normative privilege entangled within such assemblages. Whilst not raised within the workshop itself, it also provoked my thinking regarding how race and culture were entangled within my assumptions about ‘going out for coffee’ as a ‘normal’ activity of citizenship, as well as my own experiences needing to meet people for peer advocacy in coffee shops because ‘the service user’ (rather than the assemblage) had been assessed as ‘high risk’ by the clinic, and therefore ‘unsafe’ to meet in private. Attending to affect collectively challenged my thinking of spaces and practices as universally ‘safe’, ‘healing’, ‘welcoming’, or ‘oppressive’, drawing my attention instead to the diverse effects produced through shifting material–discursive relations.

### Accounting for the Material World

Our collective thinking–feeling also supported an attending to the material world as productive of such affects, pushing past the humanist centring of discourse. For example, using the image of the hospital room, we felt–thought about what, and how, matter matters. We described, for example, the following material relations that mattered within clinical spaces:

Bleach, boiled veg, body odour

The chaos of noise: alarms, running, echoing corridors

Unanswered requests at the nursing station; frustrated and angered voices

Lightness and darkness thrust upon

Hunger

Swipe cards, jangling keys

Vending machines: not working/providing comfort

Hurried staff: interrupting, gawking

Tissue box, phone, bin, bible in drawer, notepad

Chair, bed

Sanitised, sanitising

High fences, mesh

Attending to the materiality of these spaces did not mean discarding discourse but rather seeing it as entangled and attending to what emerges from such engagements. One peer, for example, reflected on how risk discourses combined with an affective atmosphere of anger, frustration, fear, and distress, the layout of clinical spaces, worker responses, and so forth meant she noticed the curtains and chairs first in the image, conducting a risk assessment and ascertaining the chairs were ‘too light’. At the same time, we talked about how the chairs provided capacity to connect with someone: “I’ve got somewhere to sit, I won’t be standing over them in a bed. Perhaps get right down to the right level.” Here, peer desires and values for connection and equality entangled with material objects to produce something other than an ‘at-risk’ subject. A chair becomes a place to connect, a weapon to harm self and others, and an object of sameness and sanitisation. The chair does not have agency of its own but rather is affective in relation with different elements.

Such entanglements – smells, objects, sounds, and time – their relations, and what they produce would not have been attended to otherwise in our thinking–feeling–activism, looked over in favour of the discursive. Together we theorised the peer “workplace[s] as an active agent among the entangled, multiple, intra-acting agents” that make up the assemblage of peer support (Van de Putte et al., 2018, p. 890), giggling as one peer worker remarked, “all the elements combined – sounds like a superhero.” For me, this moment was particularly moving, replicating the ‘pleasure and surprise in engaging with theory’ that Lather (2013) describes as “displac[ing] the fear-terror that too often characterises women’s [and Mad folks] experiences with theory-enriched data analysis” (p. 639).

### Support and Resistance as Relational

Collectively, we mapped out how entanglements of knowledge and practices, temporal rhythms, material objects, emotions, and desires produce particular experiences, identities, and practices of mental health care and peer support work. They shape how we feel–think–act towards ourselves, others, and the world around us. Intra-acting with the quotes, images, theoretical prompts, and concepts, we considered peer support as emergent within entangled material and discursive relations rather than a practice that a rational and autonomous peer support worker does to/for a passive ‘patient’ or ‘service user’. Doing so was a significant shift from what we identified as the dominant (colonial, biomedical, and patriarchal) production of care as transactional, impersonal, and ‘clear-cut’, re(enacting) workers as rational autonomous bodies acting in linear ways. Peer support became more than simply a worker acting with kindness, compassion, or competence. It became an always-becoming event or relation, realised by an assemblage of human and more than human elements (bodies, practices, spaces, discourses, and temporalities).

Considering peer support as fluid, always in flux, and always with diverse and changing effects, called us to engage with a moment-by-moment attendance to ethical practice, recognising that such effects can never be known in advance. We talked about the ‘beautiful messiness’ of supporting someone “when there aren’t answers to things … because there shouldn’t be.” Reflecting on the workshop, one peer commented:

I think I’ve just learnt more of the messiness of life …. sometimes that makes me feel a bit better because my practice does change from day to day.

Our work highlighted the heavy regulatory gaze produced through ‘inclusion’ into clinical assemblages for peer support workers, as well as moments that broke through and felt freeing. Within Australian mental health policy, peer support workers are produced as ‘change agents’ (Sinclair, Fernandes et al., 2023), responsible for moving the mental health system towards person-centred and recovery-orientated approaches. Theorising agency as the result of entangled forces meant that rather than putting responsibility for change on individual peer support workers, we were able to consider how “response-ability in the face of power imbalances” sits within a complex field of forces (Lather, 2016, p. 126). The focus shifted from whether a peer support worker resists or complies with psy-violences through ‘inclusion’ to the assemblage that produces peer support in certain ways. The effects of inclusion became multiple and complex. We reflected on being accountable in our work and also holding assemblages to account. Doing so was not only validating but also opened up “the possibility to rearrange the world through deterritorialising some aspects of it,” through multiple, emergent, small shifts (Van de Putte et al., 2018, p. 889). We were able to start considering the social, affective, material relations that enable peer support (as an event) that is more likely to produce dignity and justice that “produce wellbeing in and between bodies both human and non-human” (Duff, 2016, p. 70).

## Limitations to Our Approach and Considerations for Future Practice

Whilst bringing together post-qualitative and Mad theorising and practices sprouted new potentials for support and activism, there were also points during our collective theorising where our prior thinking–feeling was potentially reinforced. For example, whilst the visual prompts were powerful for provoking affect and thinking about materiality, our thinking–feeling did not extend much past the image to other materialities which had previously been raised in individual discussions. Health care and community settings are never static – they are always reassembling, becoming otherwise, yet an image fixes these places. Whilst this did not hamper our ability to conceptualise change and transformation, we remain curious as to what more time, prompting, different contributors, different prompts, or perhaps more active ways of considering spaces such as visiting spaces to see (feel, smell, and hear) collectively might produce (see, for example, Brice et al., 2021; Castrodale, 2018).

There also existed a tentativeness when thinking–feeling with the quotes that didn’t exist with the images. Again, multiple, entangled elements may have contributed to this; we were restricted with time, and the quotes were lengthy. Van de Putte et al. (2018) describe a collective workshop in which they “gathered together in a cosy house in the countryside for three days” (p. 890); this would be our intention for the next ethics application! I also suspect this was partly because we had not done enough to shift away from humanist thinking and thus a representational reading of the quotes. I was nervous, and I suspect others were too, about the potential to judge or critique peers, and our desires to relate or connect with the quotes. The prompts in some ways remained centred on the peer support worker as the main agent as producing peer support rather than peer support as an event produced through entanglements, a common limitation highlighted by Taguchi et al. (2020).

Further, our processes did not always disrupt the ‘sameness’ that had also limited the initial discussions. We were all white females, ‘sane enough’ to be employed by the mental health system at various points. We all knew one another and had connections to the consumer movement within Australia. These positionings led us to map assemblages in particular ways with inevitable gaps and to the exclusion of other ways. Whilst there was some discussion of socio-economic status, we did not discuss differences regarding gender, race, culture, and diagnosis. This absence however, interwoven with other provocations, including queer, black, feminist mad scholarship, and my colleagues’ prompting, has been productive to my own thinking. More time and a drawing in of additional theory would have perhaps strengthened our collective theorising in this regard.

## Bringing Together Madness and PQI: Tensions and Possibilities

We started this article with a provocation: what possibilities for new knowledges and political action might emerge by bringing PQI to (Mad) “kitchen table conversations and protests”? (Bivens, 2021, p. 399). The workshop was our experiment in doing so. It came about from journeying through a somewhat humanist approach to qualitative research, where, despite drawing on Mad theory, I became ‘stuck’. Engaging with post-humanist theories, alongside peer support workers’ experiences and practices, provided a way through, enabling a consideration of, and attending to, affect, complexity, multiplicity, and relations with the material world. The workshop, drawing on both PQI and Mad methodologies, provided a space in which we played with bringing together post-humanist theories, with a commitment to thinking critically about mental health systems and the inclusion of those with direct experience as knowledge producers and activists.

Plugging PQI into Mad/survivor research approaches supported us to move past some of the limitations of co-option and inclusion narratives. It enabled a recognition of experience as situated (as called for within the Mad studies literature by Rose (2017), Voranka (2016a) thus resisting a production of survivor/service-user experiences as authentic or universal. Instead, we attended to how we are all entangled and constantly becoming anew through ever-changing material, social, and non-human relations. It enabled us to value and work with mad feelings and mad energy for its productive potential. Envisioning peer support as an event, produced through entangled forces, opened up possibilities for activism, taking into account the broader assemblage and accounting for the complexity and multiplicity of effects.

Simultaneously, the political aims and practices of Mad research sensitised our research to injustice within mental health systems and practices of inclusion and ensured a privileging of experiential knowledges. Plugging Mad studies into PQI means that lived experience perspectives remain vital, but demands are put on research practices to enable such relations to be explored diffractively by, or in relation with, survivors/service-users themselves. This does not mean assuming that “madpersons are always already agents of liberation”; rather, it means “acknowledging that they can be” (Bruce, 2021, p. 9). Taguchi et al. (2020), who has written extensively on post-human and new materialist thinking and approaches, advise that as researchers “we need to leave the comfort of reshaping our minds with theory (as researchers) and instead reshape our minds by ways of practices in collaboration with those agents whom the research concerns” (p. 43). Collaborating with, and respecting, Mad knowledges, affects, and practices is vital to research that draws on PQI, ensuring such thinking is always attentive to justice and activism.

Thus, whilst tensions exist between emancipatory theories (such as Mad theory) and those that critique humanist and modernist thought (such as post-humanism), we take the stance here, based on our experimenting, that this tension can be productive. There already exist excellent examples of this productive potential, both within Mad studies (Aho et al., 2017; Bruce, 2021; Joseph, 2022; Tam, 2017) and critical mental health theorising (Blackman, 2015; Fullagar et al., 2019; Stone et al., 2020). We hope our work contributes to future entanglements, whereby Madness informs PQI and PQI informs Mad “kitchen table conversations and the protests” (Bivens, 2021, p. 399).
